# Mate choice in a promiscuous poison frog

**DOI:** 10.1111/eth.13331

**Published:** 2022-09-22

**Authors:** Mélissa Peignier, Lauriane Bégué, Alina Gieseke, Diana Petri, Max Ringler, Eva Ringler

**Affiliations:** ^1^ Division of Behavioural Ecology, Institute of Ecology and Evolution University of Bern Bern Switzerland; ^2^ Messerli Research Institute University of Veterinary Medicine Vienna Vienna Austria; ^3^ Department of Behavioral and Cognitive Biology University of Vienna Vienna Austria; ^4^ Department of Biology and Ecology University of Montpellier Montpellier France; ^5^ Department of Evolutionary Biology University of Vienna Vienna Austria; ^6^ Institute of Electronic Music and Acoustics University of Music and Performing Arts Graz Graz Austria

**Keywords:** Anura, call characteristics, familiarity, female mate choice, polyandry, sequential mating

## Abstract

In many animal species, members of one sex, most often females, exhibit a strong preference for mating partners with particular traits or resources. However, when females sequentially mate with multiple partners, strategies underlying female choice are not very well understood. Particularly, little is known if under such sequential polyandry females mate truly randomly, or if they actively try to spread mating events across multiple partners. In the present study, we used the highly promiscuous poison frog *Allobates femoralis* to investigate whether promiscuity could result from a preference for novel mates. Furthermore, we examined the importance of call characteristics for mate choice. We conducted mate choice experiments in a laboratory setup, by presenting females with recent mating partners or novel males. We recorded call characteristics of both males and the time females spent close to each male. In our trials, females preferred previous mating partners over novel males and also males with shorter advertisement calls. Results from previous studies on *A. femoralis* suggest that females in our trials recognized previous partners based on individual call characteristics. While mating decisions in the wild and in the laboratory might differ, our study provides first evidence for female mate choice in a poison frog with sequential polyandry.

## Graphical abstract

Females and males of the poison frog *Allobates femoralis* mate with multiple partners. We investigated the influence of call characteristics on female mating decisions, and if females avoid previous partners to actively spread reproduction across multiple partners. In our 2‐way choice experiment, females chose previous mating partners over novel ones and preferred males with shorter calls. Females likely recognize familiar males through individual call characteristics. This study provides first evidence for female mate choice in this species with sequential polyandry.

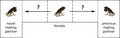

## 1 INTRODUCTION

Animals have evolved a wide variety of species‐specific mating strategies to maximize their reproductive success (Emlen & Oring, 1977; Kokko et al., 2003; Krasnec et al., 2012). Mate choice and intra‐sexual competition are key elements of many mating systems and together characterize sexual selection (Darwin, 1871; Rosenthal, 2017). In many cases, members of one sex, most often females (but see e.g. Werner & Lotem, 2003), exhibit a preference for mating partners with particular traits or resources. Females may benefit from being choosy by obtaining access to resources (e.g. nesting site, food), secure males that show high parental investment or benefit from high genetic quality or compatibility of the male that will enhance the viability in the offspring (Hamilton, 1990; Neff & Pitcher, 2005; Rosenthal, 2017; Tregenza & Wedell, 2000; Trivers, 1972). However, in populations living in highly dynamic environments, “desirable” or “optimal” traits of potential mating partners may change over time or might be difficult to assess (Bonsall & Klug, 2011). In general, females should become less choosy as costs of assessment or courtship increase. In this situation, the choosing sex may partition reproduction across multiple partners to ensure against total reproductive failure and thereby promiscuous mating might become adaptive (Carlisle, 1982; Fox & Rauter, 2003; Garcia‐Gonzalez et al., 2015; Yasui, 2001; Yasui & Garcia‐Gonzalez, 2016).

In species where females sequentially mate with multiple partners in subsequent reproductive events, strategies underlying female choice are not very well understood, and female multiple mating is often interpreted as a result of non‐choosiness by the female (Krasnec et al., 2012; Yasui, 2001). However, such a mating pattern could actually be the result of various strategies. On one hand, females may indeed opportunistically mate with available males that by chance are spatially close or advertise availability, leading to a seemingly random mating pattern (Janetos, 1980; Meuche et al., 2013). On the other hand, such promiscuous mating could be caused by females that show a preference for males they have not previously mated with (Krasnec et al., 2012; Yasui, 2001). Sequential polyandry is known to attenuate negative effects of mating with single low‐quality mates (Fox & Rauter, 2003; Yasui, 2001; Yasui & Garcia‐Gonzalez, 2016). However, whether such a mating pattern is the result of true random mating, or the active preference for novel mates remains unknown for most species with promiscuous mating systems.

To study mate choice, amphibians are a particularly interesting taxon because of their high diversity and complexity of reproductive strategies and mating systems (Luz Nunes‐de‐Almeida et al., 2021; Sullivan et al., 1995; Wells, 2007). Females of many anuran species exhibit mate choice based on characteristics of male advertisement calls (e.g. Gerhardt, 1991; Giacoma et al., 1997; Klump & Gerhardt, 1987; Schwartz et al., 2001; Tárano & Fuenmayor, 2013; Welch et al., 1998). Temporal properties of a call such as call/note duration or number of calls within a bout can convey information on a male's endurance (Taigen & Wells, 1985), on its body size (Giacoma et al., 1997) or on the quality of paternal care (Pettitt et al., 2020). Spectral properties, such as the frequency or amplitude of the call, are often linked to age (Felton et al., 2006) and body size (Humfeld, 2013; McClelland et al., 1996). However, not all anurans show clear preferences for certain traits when it comes to mate choice. Several species have evolved high levels of promiscuity, suggesting non‐selective mating by females (Roberts & Byrne, 2011).

In Neotropical poison frogs (Dendrobatidae sensu AmphibiaWeb, 2021), female mate choice is mostly based on visual and acoustic signals of potential mating partners. Visual cues predominantly play a role in the colourful, aposematic species, where dorsal brightness, spectral reflectance and colouration patters often enable assortative mating (e.g. in *Oophaga pumilio*, Dreher et al., 2017; Gade et al., 2016; Maan & Cummings, 2008, 2009, 2012; Reynolds & Fitzpatrick, 2007; Richards‐Zawacki et al., 2012; Richards‐Zawacki & Cummings, 2011; Summers et al., 1999). However, the visually mediated assortative mating in *O. pumilio* has recently been shown to become overridden by the outcome of direct male–male competition. In the wild, female *O. pumilio* prefer to mate with males of the local colour morph. A study showed that this preference is overridden by intra‐sexual selection, as females tested in a laboratory setting preferred territory holders over non‐territorial males, regardless of their colour morph (Yang & Richards‐Zawacki, 2020). Acoustic signalling is present in the vast majority of poison frogs across all clades, with different acoustic characteristics being relevant for female mate choice (e.g. Dreher & Pröhl, 2014; Lüddecke, 2002; Meuche et al., 2013; Pettitt et al., 2020; Pröhl, 2003; Roithmair, 1994; Souza et al., 2021). Also territory size and breeding resources, which the female can assess during courtship within the male's territory, seems to play a role in female mating decisions (e.g. in *Allobates paleovarzensis* [Da Rocha et al., 2018], *Ameerega trivitatta* [Roithmair, 1994], *Oophaga pumilio* [Donnelly, 1989; Pröhl & Hödl, 1999]).

In the Brilliant‐thighed Poison Frog *Allobates femoralis* (subfamily Aromobatinae sensu AmphibiaWeb, 2021), both sexes typically mate multiple times and with different partners, resulting in a highly promiscuous mating system (Montanarin et al., 2011; Stückler et al., 2019; Ursprung et al., 2011). Previous studies point towards the absence of active mate choice by females because of high levels of polyandry and low levels of reproductive skew among males (Ringler et al., 2012; Ursprung et al., 2011). Female preference for males with larges territories was described in an observational study by Roithmair (1992); however, these findings were not corroborated in a more recent study using molecular methods to measure reproductive success (Ursprung et al., 2011). So far, the possibility of an active preference for novel mating partners has not been studied and previous studies did also not incorporate male call characteristics into analyses of female choice. Given the substantial energetic costs and risks of predation that male frogs generally face when calling, calls could be expected to serve as a signal of male quality, directed at and used by females (Pough & Taigen, 1990; Ryan et al., 1982, 1983; Taigen & Wells, 1985; Zahavi, 1977). Furthermore, previous studies have shown that the calls of *A. femoralis* males are individually distinct which could help females identify and favour novel partners for mating (Gasser et al., 2009; Tumulty et al., 2018).

To investigate patterns of female mating in *A. femoralis*, we conducted a choice test where we presented a previous mating partner and a novel male to females in a two‐arm maze. We made the following predictions:

1. if females prefer novel mating partners, they should spend more time close to a novel male compared with in the central section or close to their previous mating partner;
2. if females prefer males based on the amount and/or characteristics of the advertisement or courtship call, female choice should be correlated with the number, the duration, the frequency and/or the consistency of male calls.

## 2 MATERIALS AND METHODS

### 2.1 Study species

**FIGURE 1:**
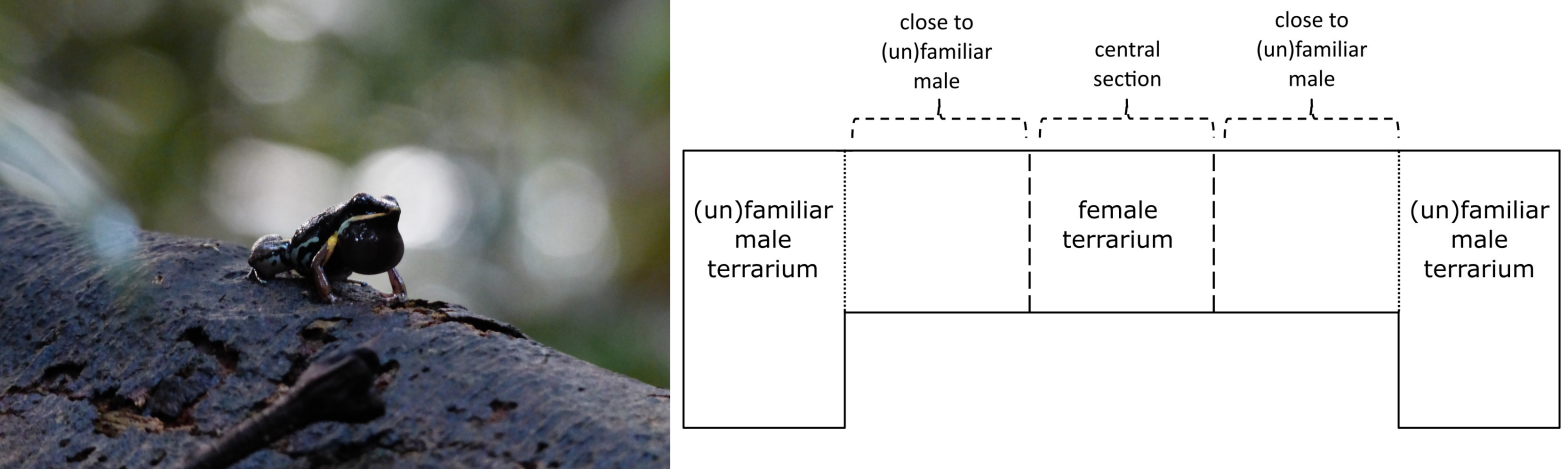
Picture of a male *Allobates femoralis* calling (left) and scheme of the two‐arm maze (right). The setup consisted of a middle terrarium (120 × 35 × 70 cm) housing the female and two adjacent terraria (60 × 35 × 70 cm) housing a previous mating partner and a novel male. The dashed lines represent the white markers used to divide the females' terrarium into three thirds for the analyses. The middle and adjacent terrarium were separated with sliding doors made of glass on the lower half and mesh on the upper half (dotted lines).

*Allobates femoralis* (Figure 1) is a Neotropical poison frog that occurs commonly throughout the Amazonian basin and Guiana shield (Amezquita et al., 2009). During the reproductive season, which coincides with local rainy seasons (Gottsberger & Gruber, 2004), males are highly territorial and advertise territory occupancy via prominent advertisement calls from elevated perches to repel male competitors and attract female mating partners (Hödl et al., 2004; Ringler et al., 2011). Advertisement calls consist of four notes which feature an upward frequency sweep, with calls being repeated at regular intervals to form bouts of up to 40 four‐notes calls (Narins et al., 2003). Females do not establish territories but show site fidelity and commute to male territories within 20 m for courtship and mating (Fischer et al., 2020; Ringler et al., 2012). Males switch from advertisement calls to courtship calls once a female is in sight. The buzzing courtship call lasts for .5–1 s and features a broadband burst of pulses with a dominant frequency of 2500–2700 Hz (see Figure 1 in Stückler et al., 2019). Once the male switches to courtship calling, the pair begins an elaborate courtship sequence that can last up to several hours and almost always end up in successful oviposition (Montanarin et al., 2011; Stückler et al., 2019). The courtship march in *A. femoralis* is among the longest observed in poison frogs and likely serves the pair to identify a suitable oviposition site or collect fine‐scale spatial information needed for subsequent parental care (Stückler et al., 2019). The female lays a clutch of approximately 20 eggs in the leaf litter, which is then externally fertilized by the male, and subsequently the female leaves the male's territory (Ringler et al., 2015). After 15–20 days of larval development, tadpoles hatch and are transported by the male to medium‐sized natural pools located up to 200 m from their territory (median: 27.52 m ± 30.90 m iqr; Ringler et al., 2013; see also Beck et al., 2017; Ringler et al., 2018). Males partition tadpoles of single clutches over several water bodies in an attempt to hedge their bets regarding parental care (Erich et al., 2015; Pašukonis et al., 2017). Occasionally females take over transport, but only when the father is absent (Ringler et al., 2015).

### 2.2 Housing conditions

We performed the behavioural experiments under controlled laboratory conditions from October to December 2019 in the animal care facilities at the University of Vienna using individuals from a captive *A. femoralis* population. Prior to the choice experiments, we kept the frogs in randomly assigned pairs in standard glass terraria of equal size (60 × 40 × 40 cm) with identical equipment and furnishings. The floor was made of expanded clay pebbles, back and side walls were covered with Xaxim (tree fern stems) mats in the lower and cork in the upper half to prevent visual contact between terraria. Half a coconut shell, a small plant, and a branch provided standardized shelter and elevated calling positions. We provided autoclaved oak leaves as substrate for oviposition, and a small glass bowl of 10 cm diameter filled with water for tadpole deposition. An automatic raining, heating and lighting system ensured standardized climatic conditions in all terraria, similar to natural conditions in French Guiana. The temperature ranged from 21°C at night to 28°C during the day. Lights were on from 7 a.m. to 7 p.m. and humidity in the terraria was constantly at 100%. Frogs were fed wingless fruit flies (*Drosophila*) three times a week but were never fed on the day of an experiment. Pairs used in our experiment had already been kept together over several months, and each pair had already produced clutches together. We also made sure that the novel male and the female used in a test had not been in contact (i.e. housed in the same terraria) before.

### 2.3 Mate choice test setup

We performed the mate choice test in a separate room using a two‐arm maze. The middle area (120 × 35 × 70 cm) housed the female, while the two adjacent areas to the left and right (60 × 35 × 70 cm each) housed the previous mating partner from her home terrarium, and a novel male she had never had direct contact with, respectively (Figure 1). The side areas housing the males were separated from the middle part with sliding doors made of glass on the lower half and mesh on the upper half to allow the female to see, smell and hear both males. All area had expanded clay pebbles as soil material, which was covered with autoclaved oak leaves. We sprayed and rinsed each compartment with 2 L of demineralized and dechlorinated water before each trial to maintain an equal level of humidity for each trial and remove odour cues from the previous trial. The light and heat conditions were the same as the ones provided in the housing room. The males were provided with half a coconut shell for shelter and a cork branch for calling. In the female's compartment, we placed small white markers on the ground to divide the area into three thirds (corresponding to “close to previous partner,” “central section,” “close to novel male”).

### 2.4 Mate choice test procedure

To ensure that females were physically ready to produce a new clutch and likely motivated to find a mate, we waited a minimum of 6 days after their last clutch to perform the test (cf. Weygoldt, 1980; in captivity on average 8 days between oviposition). We tested each female only once, while males could serve as the previous partner and novel male in one trial each. To control for a possible directional bias of females in the setup, we alternated the side where we presented previous partner/novel male. We also controlled for the effect of male body size by choosing a novel male of similar stature to the previous partner. All frogs had at least 60 min to acclimate to the testing terraria before the experiment started. Females could see, hear and smell both males during this acclimation period. We then recorded the behaviours of each female for 180 min between 3 p.m. and 6 p.m. with digital full‐HD surveillance cameras (BX400 HD Minidome; IndigoVision) that were installed on top of the setup. Additionally, we recorded male calls with two Lavalier microphones (TY‐109; renkforce) attached to pre‐amplifiers (PS418S; Superlux) and a digital audio recorder (24‐bit, 44.1 kHz; H4N, Zoom). Microphones were inserted into the male compartments to record calls during trials. After the trial, we photographed each individual on millimetre paper to measure snout‐urostyle length (SUL) with ImageJ (Rasband, 1997–2021), before transferring them back to their home terraria. We conducted 23 trials in total, one trial per female.

### 2.5 Data collection

For coding the location of females in the video recordings of the trials we used the software BORIS (Friard et al., 2016). To determine whether females prefer a previous partner or rather a novel male, we analysed the relative duration a female spent close to each male without taking into account the time spent in the central section (hereafter “proportion of paired time”). The proportion of paired time was calculated by dividing the time spent near a particular male (previous partner or novel male) by the total time spent near either male within a trial. Additionally, we coded for each male whether the female chose him (1) or not (0) during the test. We coded a male as chosen if the female spent more than half of the proportion of paired time with him.

We used the bioacoustics software RAVEN PRO 1.6.1 (K. Lisa Yang Center for Conservation BioacousticsYang Center for Conservation Bioacoustics, 2011–2021) to determine the spectral and temporal characteristics of the advertisement and courtship calls. We counted the total number of advertisement (i.e. four‐notes call) and courtship calls. Then we applied a band energy detector to find entire bouts of advertisement calls (detector settings: minimum frequency: 2000 Hz; maximum frequency: 5000 Hz; minimum duration: 1 s; maximum duration: 50 s; minimum separation: 2 s; minimum occupancy: 14%; SNR threshold: 29 dB; block size: 5 s; hop size: .1 s; percentile: 30). We further applied additional band energy detectors to find single *A. femoralis* advertisement and courtship calls (detector settings: minimum frequency: 2000 Hz; maximum frequency: 5000 Hz; minimum duration: .3 s; maximum duration: 1.7 s for advertisement calls and 3.0 s for courtship calls; minimum separation: .27 s for advertisement calls and .01 s for courtship calls; minimum occupancy: 40% for advertisement calls and 75% for courtship calls; SNR threshold: 29 dB for advertisement calls and 13 dB for courtship calls; block size: 5 s; hop size: .1 s; percentile: 30). Finally, to find the four separate notes constituting the advertisement calls, we applied another band energy detector (detector settings: minimum frequency: 2000 Hz; maximum frequency: 10000 Hz; minimum duration: .03 s; maximum duration: .2 s; minimum separation: .01 s; minimum occupancy: 10%; SNR threshold: 29 dB; block size: 5 s; hop size: .1 s; percentile: 30) on the cut‐out files of the detected bouts. From the automatic detections, we used the mean duration (s) and mean peak frequency (Hz) of the four‐note advertisement calls and of the courtship calls. Then, we calculated the advertisement call rate as the number of calls in a bout per min. We also calculated the coefficients of variation within males of the time interval between the first and second, second and third, and third and fourth notes (i.e. standard deviation divided by the mean). Likewise, we calculated the coefficient of variation of the inter‐call interval (i.e. time between the last note of a call and the first note of another call within a bout). Finally, we calculated the coefficient of variation of the frequency range and of the mid‐frequency of each note. We coded males that did not call with a call number, call rate and a mean call duration of 0, and “NA” for the other measurements.

### 2.6 Statistical analyses

We conducted all statistical analyses in R v3.6.0 (R Core Team, 2020), using the integrated development environment RStudio v1.3.1093 (RStudio Team, 2019). First, we verified that we used males of similar size by comparing the size of the previous partner and novel male using an independent two samples *t*‐test. We checked data for normal distribution of each group with a Shapiro–Wilk test (size of the previous partner: statistic = .944, *p*‐value = .223; size of the novel male: statistic = .948, *p*‐value = .266) and for homogeneity of variance with Levene's test (df1 = 1, df2 = 44, statistic = .609, *p*‐value = .439). We also checked for a potential directional bias in females. We checked for data normality (Shapiro–Wilk test: statistic = .822, *p*‐value <.001) and used a Wilcoxon one sample test comparing the proportion of paired time (whether it was with the previous or novel male) spent on the left side to .5. As both tests did not reveal significant differences in body size or any side bias across tests, we did not include these variables in any further analyses.

Next, we investigated whether females were responsive to the presence of males. We built three Wilcoxon one sample tests comparing the proportion of time spent in each section to .3333, which represents a third of the total trial time. In other words, we investigated whether females spent more or less than 1/3 of the time in each section. Here, we used non‐parametric tests because the time spent in each section did not follow a normal distribution (Shapiro–Wilk test: proportion of time close to the novel male: statistic = .839, *p*‐value = .002; proportion of time close to the previous partner: statistic = .862, *p*‐value = .004; proportion of time in the central section: statistic = .740, *p*‐value < .001). Then, we analysed the female's preference for a male based on familiarity by using a Wilcoxon one sample test to compare the paired proportion of time spent close to the novel male to .5. Again, we used a non‐parametric test because the response variable did not follow a normal distribution (Shapiro–Wilk test: statistic = .811, *p*‐value = .001).

We investigated the influence of different characteristics of the advertisement and courtship call on female choice behaviour. We built three generalized linear mixed effect models (GLMMs) following a binomial distribution with the function “glmer” in the lme4 package (Bates et al., 2016). We used the choice of the female (if the female spent most of the time with the male −1‐ or not −0‐) as a response variable in all three models. In the first model, we added the coefficients of variation of the inter‐notes intervals, inter‐call interval, frequency ranges of each note, mid frequencies of each note and the mean peak frequency of calls as fixed effects. All fixed effects were scaled using the “scale” function in R (i.e. centred to their mean value and standardized to units of 1 phenotypic standard deviation). In the second model, we added the number and duration of advertisement and courtship calls, and the rate of advertisement calls as fixed effects. All fixed effects were scaled. In the last model, we added the mean frequency of courtship calls as a fixed effect. In all three models, we included trial as a random effect. We built several separate models instead of one full model with all parameters to make use of the maximum amount of data in each model. For instance, since not all individuals performed courtship calls, including the mean peak frequency of courtship calls in the model with the number and duration of calls would have led to a loss of data.

Both the familiarity and the call duration seemed to influence the time a female spent close to a male (i.e. females spent more time close to their previous partner and close to males whose advertisement call was shorter; see “Results” for more details). Therefore, we further investigated whether this could be the result of males producing shorter calls for females they already know and have mated with. To test whether there is a link between call duration and familiarity, we fitted a linear model with the call duration as response variable and the status of the male (previous partner vs. novel male) as a fixed effect. We added trial and male ID as random effects. We applied a constant transformation on the call duration using the function transformTukey and inspected model residuals for normal distribution using diagnostic qq‐plots. We acknowledge that introducing an interaction between familiarity status and call characteristics would have been particularly interesting to deepen our understanding of female choice behaviour, but unfortunately our sample size does not allow for such analysis.

### 2.7 Ethical note

This study was approved by the ethics and animal welfare committee of the University of Vienna (No. 2019‐003) in accordance with Good Scientific Practice (GSP) guidelines and national Austrian legislation. The frogs used in this experiment belonged to an ex situ laboratory population at the animal care facility of the University of Vienna. Original stock for this population, including all animals used for this study, was sampled in and exported from French Guiana in compliance with all legal requirements from the responsible French authorities (DIREN: Arrêté n°82 du August 10, 2012 and Arrêté n°4 du January 14, 2013). We followed the guidelines laid out by the ASAB for the treatment of animals in behavioural research and Teaching (Asab, 2020) and the ARRIVE guidelines (Percie du Sert et al., 2020).

## 3 RESULTS

We first confirmed that we used novel males and previous partners of similar size (independent two samples *t*‐test, *t*‐value = −1.031, *p*‐value = .308). We also verified the absence of a potential side bias on female choice (Wilcoxon one sample test, V = 162, *p*‐value = .472).

**FIGURE 2:**
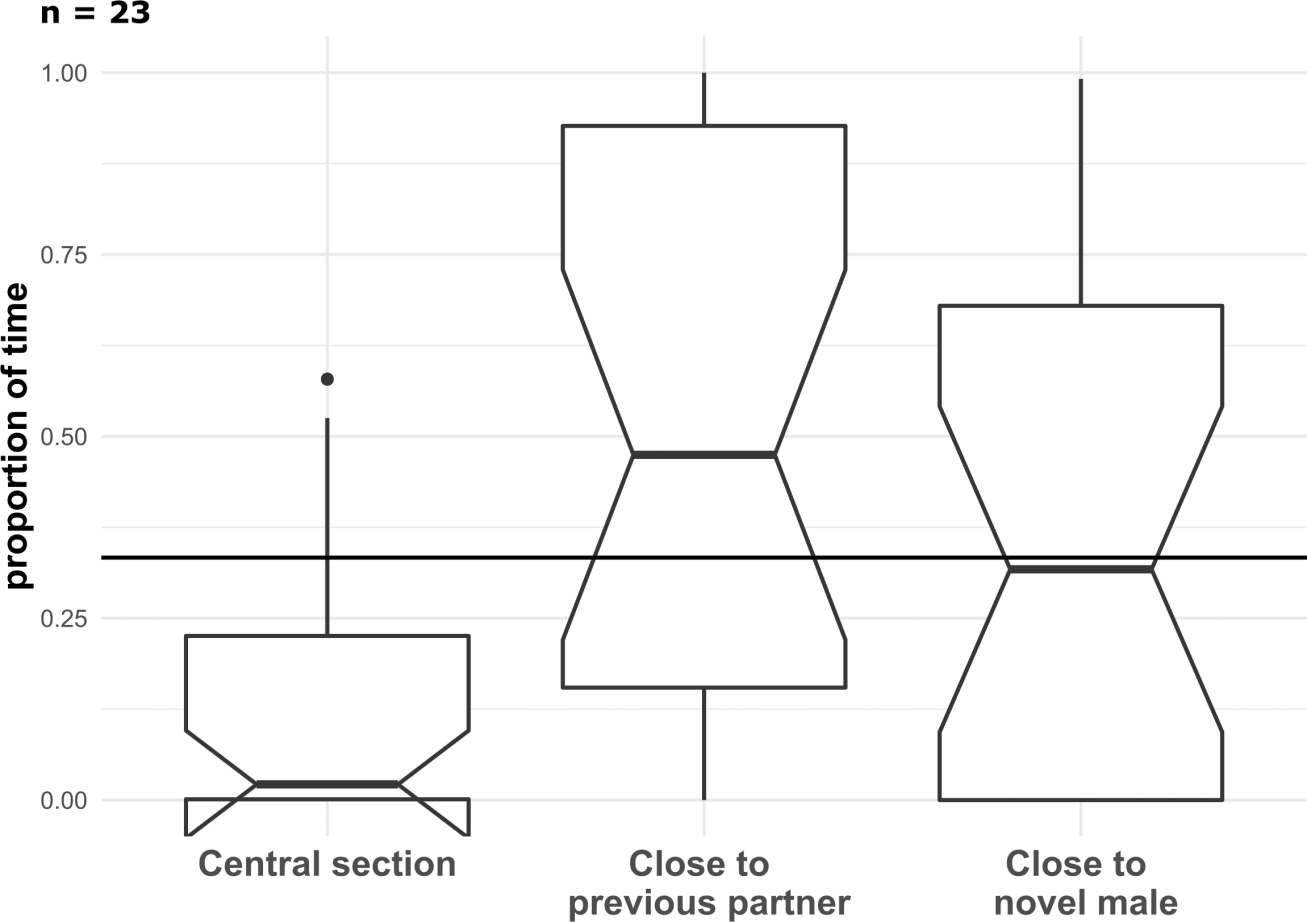
Boxplot of the proportion of time spent by females in each section. The horizontal line crossing the entire plot represents a third of the total time. The median is represented by thick dark lines, the interquartile range is represented by the upper and lower edges of each box, the qualitative difference in median is represented by the notches, and the upper and lower quantiles (1.5 × IQR) are represented by the whiskers.

When testing for a preference based on familiarity, we first checked that females were responsive to the presence of male. In average females spent 50% of their time close to the previous partner, 35% close to the novel male and 15% in the central section. We found very strong evidence (sensu Muff et al., 2021) that females spent significantly less than 1/3 of the time in the central section (*z* = −3.35, *V* = 28, pseudo median = .09, *p*‐value <.001), while we found weak evidence that they spent more than 1/3 of the total trial time close to the previous partner (*z* = 1.83, *V* = 198, pseudo median = .51, *p*‐value = .07; Figure 2). Finally, females spent around a 1/3 of the time close to the novel male (*z* = .34, *V* = 149, pseudo median = .34, *p*‐value = .749; Figure 2). We also found that females did not spend more or less than half of the total paired time close to the novel male (*z* = −1.19, *V* = 99, pseudo median = .44, *p*‐value = .238).

**FIGURE 3:**
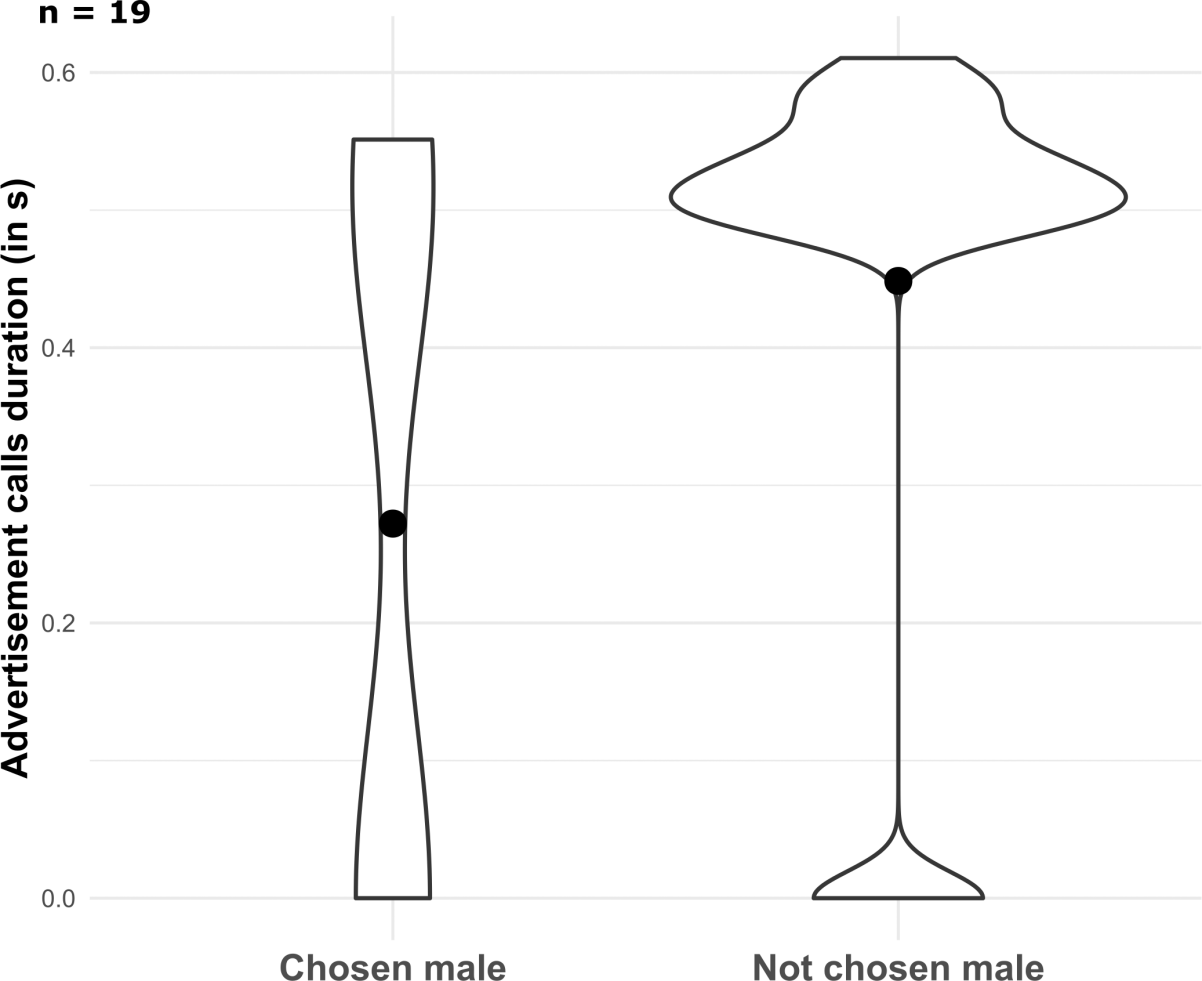
Violin plot of the call duration (in seconds) of the chosen and non‐chosen males (i.e. males with whom the females spent respectively more and less than half of the paired proportion of time). The dot represents the mean.

When investigating the effect of call characteristics on female choice, our model results showed moderate evidence that females spent more time close to the males whose advertisement calls were on average shorter (Figure 3; Table 1) and we found weak evidence that females spent more time close to the males who called at a higher rate (Table 1). The amount and average frequency of calls had no effect on female's choice (Table 1). The consistency of the time interval between two notes of the frequency range and mid frequency of the notes did not influence female's choice (Table 1). None of the measured courtship call characteristics had an effect on female choice (Table 1). Based on our results, we wondered if males possibly produced shorter calls for the females they have already mated with (i.e. previous partners). However, we did not find a link between call duration and male familiarity status (Table 1). Call duration of previous partner and novel males did not differ, which confirms that call duration in itself is an important factor in female mate choice, no matter if the female already mated with the calling male or not. To sum up, in our trials females preferred previous mating partners over novel males. They also preferred males with shorter advertisement call, but previous partners and short‐calling males were not necessarily the same individuals.

**TABLE 1:** Results of the generalized linear mixed effect models looking at how call characteristics of male advertisement and courtship calls affect female choice behaviour

Response variable	Fixed effects	*N*	Estimate	Standard error	*p*‐Value
Female's choice	Intercept	24	−1.05	.98	.285
	Time interval note 1 – note 2		−2.06	1.77	.246
	Time interval note 2 – note 3		.47	1.56	.763
	Time interval note 3 – note 4		1.48	1.55	.340
	Time interval between calls		−2.83	1.81	.119
	Frequency range note 1		1.35	2.38	.570
	Frequency range note 2		1.34	3.52	.703
	Frequency range note 3		−2.34	3.45	.498
	Frequency range note 4		−.34	1.55	.829
	Mid frequency note 1		.91	2.14	.670
	Mid frequency note 2		−1.76	2.56	.491
	Mid frequency note 3		2.72	3.84	.480
	Mid frequency note 4		−.63	2.97	.833
	Advertisement call frequency		1.91	2.79	.494
	**Random effects**				
	Trial		0	0	
Female's choice	Intercept	34	.01	.44	.984
	Number of advertisement calls		.30	.54	.577
	Advertisement calls duration		−4.75	2.10	**.024**
	Advertisement calls rate		3.52	1.97	**.074**
	Number of courtships calls		.70	.57	.220
	Courtships calls duration		−.53	.58	.360
	**Random effects**				
	Trial		0	0	
Female's choice	Intercept	20	5.58	4.14	.178
	Courtship call frequency		−.00	.00	.194
	**Random effects**				
	Trial		0	0	
Advertisement call duration	Intercept	38	.02	.00	<.001
	Status (previous partner vs novel)		.00	.00	.436
	**Random effects**				
	Male ID		1.89 × 10^−4^	.01	
	Trial		.00	.00	
	Residual		5.58 × 10^−5^	.01	

*Note*: Sample size (*N*) are presented for each model. Results indicating at least weak evidence (sensu Muff et al., 2021) are written in bold. The fixed effects in the first two models have been scaled (i.e. centred to their mean value and standardized to units of 1 phenotypic standard deviation).

## 4 DISCUSSION

In the present study, we investigated mechanisms underlying female mate choice in the Neotropical poison frog *Allobates femoralis*. Previous studies in this species had revealed a highly promiscuous mating system, suggesting low opportunity for sexual selection and a seemingly random mating strategy in females (Montanarin et al., 2011; Ringler et al., 2012; Stückler et al., 2019; Ursprung et al., 2011). Yet, a preference for novel mating partners by females, or a choice based on the characteristics of male advertisement or courtship calls had not been investigated and therefore could not be ruled out so far. Here, we show that females tested in the laboratory in a two‐choice test seem to have a preference for their previous mating partner over novel males and also for males (previous partners or not) with shorter 4‐note advertisement calls.

We did not find a significant difference in the time females spent with the previous versus the novel partner, yet we found a trend towards a preference for their previous partner. Females spent about half of the time close to their previous partner against 35% of the time close to the novel male. Our initial expectation that females would prefer novel males was motivated by potential benefits associated with multiple mating partners (Evans & Magurran, 2000; Hosken & Blanckenhorn, 1999; Jennions & Petrie, 2000). In our captive colony, females had been cohabitated with the respective previous mating partners in a restricted space (i.e. terrarium) for a relatively long time prior to the experiment. We cannot rule out that these particular housing conditions might have induced habituation and/or caused pair bonding and thereby influenced the decision to stay with these males in our choice experiment. However, although pair bonding and even monogamy have been observed in other poison frogs (Brown et al., 2010; Caldwell, 1997), this behaviour is unknown from the field in *A. femoralis*. The long cohabitation prior to the experiment also means that all females had previously witnessed successful reproduction with, and beneficial paternal care provided by the respective male. Therefore, choosing the previous partner might have been the females' most promising way to ensure future reproduction based on the knowledge gained from previous experience.

As another possibility, in direct contests female *A. femoralis* might prefer known territory holders over males with unknown territorial status, similar to observations in the related poison frog *Oophaga pumilio*, where direct male–male contest overrules a strong tendency for assortative mating by colour morph (Yang & Richards‐Zawacki, 2020). In our experimental setup, females only had information about the territorial status of the familiar male, whereas the status of the new male was unknown to them. We also cannot rule out that females had made their choice already prior to being in the experimental setup. All females needed to be ready to mate at the time we transferred them into the setup, which we decided based on the time since their last clutch. Therefore, they could have already decided to mate with the only available male at the time before the experiment, which was the previous partner, before we gave them the option of another, novel male during the experiment. However, in our opinion it is unlikely that this had been the case for the majority of females tested in our experiment.

In the wild, females *A. femoralis* do not stay in close proximity to males but rather commute from their resting perches to male territories for mating (Fischer et al., 2020; Ringler et al., 2012). After oviposition, they do not stay long enough to directly witness and assess the parental abilities of the male (Fischer et al., 2020; Stückler et al., 2019). Therefore, under natural conditions females are unable to predict benefits that might arise from actively choosing to mate again with a previous partner. Preliminary data and long‐term monitoring of a wild population show that, in low‐density areas or isolated situations, females mate repeatedly with the same male (E. Ringler, unpublished data). In these areas, females probably trade‐off the potential benefits of multiple mating partner against the costs to search for and access them and decide for an apparently monogamous mating strategy. We see the possibility that, in our experiment, where we presented only two males to the female, this condition resembled such a low‐density situation.

Although we cannot rule out that visual recognition also played a role in our experiment (e.g. via the ventral colour pattern or the shape of the lateral bright line), we assume that females recognized previous partners based on their call characteristics. Previous research has shown that the calls of *A. femoralis* males are individually distinct and would allow for individual discrimination (Gasser et al., 2009; Tumulty et al., 2018). In our captive breeding colony, all *A. femoralis* were housed together as pairs in separate tanks but in the same room. In this setting, males from the other tanks were strongly attenuated and audible only as a conflated background chorus. Each female was therefore more subjected to the calls of the male she was housed with, making it more likely for her to learn and subsequently discriminate distinctive features of his call. In the wild, females commute from their resting site to males' territories within 20 m and typically make the decision on whom to mate with before approaching a male (Stückler et al., 2019). Therefore, it is unlikely that females would choose a mating partner based on familiar morphological features or chemical stimuli. Recognition based on call acoustic cues is much more plausible.

In the present study, we also investigated whether female choice is based on certain characteristics of the advertisement or courtship call. Given the costs associated with advertising in terms of energy expenditure and elevated risk of predation (Ryan et al., 1982; Wells, 2001; Zahavi, 1977), call characteristics in anurans may convey important information about overall male quality. We found that the acoustic characteristics of courtship calls did not affect female behaviour in our experiment. As male *A. femoralis* only switch to courtship calls when the female is visible and in the immediate vicinity, this result is in line with previous observations in the field, that females make their mating decisions before approaching males and engaging in close distance courtship (Stückler et al., 2019).

Our results also show that females prefer the male whose advertisement calls were shorter on average, whether they were previous mating partners or not. Advertisement calls became shorter or longer by a change in the note and inter‐note interval duration. We also found weak evidence that females prefer males with higher advertisement call rates. In numerous anuran species, spectral and temporal call properties are used by females to assess the quality of potential mates (Gerhardt, 1991; Giacoma et al., 1997; Klump & Gerhardt, 1987; Schwartz et al., 2001; Tárano & Fuenmayor, 2013; Welch et al., 1998). For example, call duration is often correlated with male body size (e.g. in the green toad *Bufotes viridis*: Giacoma et al., 1997). However, since we controlled for equal body size of the two presented males in our experiment, we see no evidence that the choice for shorter calls could be related to a preference for smaller males. Producing short calls requires a quick adjustment of the muscles involved in the production of calls and therefore could be a reliable indicator of high motivation, better cognitive performance and/or motor control in calling males (Prestwich, 1994). Likewise, to call at a higher rate requires more power and therefore could lead to a higher energy expenditure, potentially representing an honest signal of male quality. A recent study also found a preference of females for males with a higher call rate in the Cope's gray treefrog (*Dryophytes chrysoscelis*). However, this preference decreased if the timing of individual calls became increasingly inconsistent (Tanner & Bee, 2020). In our experiment, the consistency of the males' calls in the temporal and spectral domain did not influence female choice. Future studies should look at the energy expenditure in the production of different components in the advertisement call.

Advertisement calls have also been shown to signal the quality of paternal care in another dendrobatid frog, the Golden Rocket Frog (*Anomaloglossus beebei*). Males who produced longer calls also provided higher quality care and were preferred by females (Pettitt et al., 2020). So far, we do not have any indication that advertisement calls in *A. femoralis* convey information about male parental state or that parental males are actually preferred by females. But to investigate the relationship between male care, call characteristics and female preferences might be an interesting topic for future studies.

While mating decisions in the wild and in the laboratory might differ, our study provides first evidence for active female mate choice in a poison frog with sequential polyandry. One aspect that a two‐choice test fails to address is the availability of further males, as it is the regular situation in the wild, and which could lead the females to make less than optimal choices as a result of a decoy effect. Lea and Ryan (2015) found that Tungara Frogs (*Physalaemus pustulosus*) subjected to two males choose the one with the most appealing call (i.e. with low dominant frequency, longer durations and faster call rates in their study) but reverse their choice when a third male with the least appealing call is introduced. Although we found evidence for choice being based on certain characteristics of the advertisement call produced by males, further studies are needed to investigate the link between characteristics of advertisement calls, mate choice and reproductive success in *A. femoralis*. In other dendrobatid species, a link between female mate choice and overall calling activity of individual males has been found (Pröhl, 2003; Roithmair, 1994; Souza et al., 2021). Future studies in the wild should investigate whether female choice in *A. femoralis* is based on immediate call characteristics or on the accumulated information on long‐term calling effort and acoustic interactions between advertising males. Our study thereby provides a basis for further studies into female mate choice in *A. femoralis*.

## AUTHOR CONTRIBUTIONS

**Mélissa Peignier:** Conceptualization; methodology; formal analysis; investigation; writing – original draft; writing – review and editing; visualization; project administration. **Lauriane Bégué:** Methodology; formal analysis; investigation; writing – original draft; writing – review and editing. **Alina Gieseke:** Methodology; formal analysis; investigation; writing – original draft. **Diana Petri:** Methodology; investigation; formal analysis; writing – original draft. **Max Ringler:** Methodology; resources; writing – review and editing. **Eva Ringler:** Conceptualization; methodology; resources; writing – review and editing; supervision; project administration; funding acquisition.

## Data Availability

The datasets generated during and/or analyzed during the current study are available in the Open Science Framework repository: https://doi.org/10.17605/OSF.IO/U82YH.
